# RNAi-directed knockdown in the cnidarian fish blood parasite *Sphaerospora molnari*

**DOI:** 10.1038/s41598-024-54171-0

**Published:** 2024-02-12

**Authors:** Jiří Kyslík, Ana Born-Torrijos, Astrid S. Holzer, Anush Kosakyan

**Affiliations:** 1 Institute of Parasitology, Biology Centre, Academy of Sciences of the Czech Republic, Ceske Budejovice, Czech Republic; 2 Department of Coastal Systems, NIOZ Royal Netherlands Institute for Sea Research, Den Burg, PO Box 59, 1790 AB Texel, The Netherlands; 3 Fish Health Division, University of Veterinary Medicine, Vienna, Austria; 4 Department of Life Sciences, University of Modena and Reggio Emilia, Modena, Italy; 5 National Biodiversity Future Center (NBFC), Palermo, Italy

**Keywords:** Molecular biology, RNAi

## Abstract

RNA interference (RNAi) is an effective approach to suppress gene expression and monitor gene regulation. Despite its wide application, its use is limited in certain taxonomic groups, including cnidarians. Myxozoans are a unique group of cnidarian parasites that diverged from their free-living ancestors about 600 million years ago, with several species causing acute disease in farmed and wild fish populations. In this pioneering study we successfully applied RNAi in blood stages of the myxozoan *Sphaerospora molnari*, combining a dsRNA soaking approach, real-time PCR, confocal microscopy, and Western blotting. For proof of concept, we knocked down two unusual actins, one of which is known to play a critical role in *S*. *molnari* cell motility. We observed intracellular uptake of dsRNA after 30 min and accumulation in all cells of the typical myxozoan cell-in-cell structure. We successfully knocked down actin in *S*. *molnari *in vitro*,* with transient inhibition for 48 h. We observed the disruption of the cytoskeletal network within the primary cell and loss of the characteristic rotational cell motility. This RNAi workflow could significantly advance functional research within the Myxozoa, offering new prospects for investigating therapeutic targets and facilitating drug discovery against economically important fish parasites.

## Introduction

Post-transcriptional gene silencing elicited by the conserved cellular mechanism of RNA interference (RNAi), common in eukaryotes, represents a ubiquitous reverse-genetic application for studying gene function in biology. Several effector molecules are used to trigger the rapid degradation of target gene messenger RNA (mRNA) to regulate gene expression. By systematic delivery (e.g., electroporation, injection, soaking, etc.) of designed small interfering RNAs (siRNA) or double-stranded RNA (dsRNA), gene expression can be regulated in a targeted manner^1^. However, the non-specific, off-target and downstream effects of siRNAs can result in various changes in gene expression^2^ and immune responses^3^ via binding of unintended mRNAs that interfere with successful gene silencing. Due to the varying effectiveness of short interfering molecules in efficient gene silencing, soaking with double-stranded RNA (dsRNA) is a commonly applied delivery method^4–8^, which can overcome specificity issues reported in siRNA-based gene silencing studies^9,10^. However, using dsRNA in gene knockdown requires parameter optimization and evaluation regarding the target organism of use^11^. RNAi studies are demanding in non-model organisms that are difficult to cultivate in vitro, have complex life cycles, or are inappropriate for delivery methods for other reasons^12–14^. Among these, cnidarian parasites belonging to the Myxozoa represent a challenging model. These microscopic organisms with a simplified body plan explicitly form typical cell-in-cell formations consisting of primary cells containing secondary cells, which contain tertiary cells^15^, similar to a Russian doll model. This species-rich group of endoparasitic cnidarians possesses distinct structures for cell motility and adapted various infection mechanisms and parasitic strategies^16–19^. RNAi-mediated knockdown (KD) has only once been performed in myxozoans, by soaking of oligochaetes harboring *Myxobolus cerebralis*^20^, however, no study has yet targeted myxozoan developmental stages directly. To do so, parasite isolation from host cells and in vitro culture is a prerequisite, which is missing for Myxozoa mainly due to the drawback of their endoparasitic life strategy and, therefore, challenging extraction from their hosts^21^. Several studies have attempted the establishment of in vitro cultures of Myxozoa^22–25^. Neither long-term persistence nor viability was observed^22^. However, most recent advances in our laboratory achieved pure parasite isolation and short-term culture^26^.

The myxozoan *Sphaerospora molnari*, which infects common carp (*Cyprinus carpio*), represents a unique model as it belongs to a genus that forms proliferative blood stages with cell “twitching” movement^27,28^. This uniquely evolved non-directional self-axial cell rotation, named Membrane Fold Induced Tumbling motility (MFIT), is generated by spatially distributed membrane folds of the primary cell with rapid reabsorption and re-establishment^28^. A derived beta actin (*ACT1*), localized in the cytoplasm, membrane, and folds of the primary cell, has been identified as the molecular driver of this unique cell behavior^28^. Besides evolutionary conserved beta actins, which have a fundamental role in cellular processes, parasites evolved actin molecules with multiple functions related to invasion, cell migration, host evasion^29^. Recent findings have highlighted the dispensable role of myxozoan beta actins in cytoskeleton dynamics, cell motility, and spore stage creation, as identified in proteomic data of *Ceratonova shasta*^30^. Compared to the classical fibrillar actin (F-actin) of the cytoskeleton used for adhesion and motility in *C. shasta*^18,19^, *S. molnari* displays short actin filaments with negative staining effect for custom phalloidin due to a derived gene sequence characteristic^28^. Besides multicellular Myxozoa, unicellular parasites of the phylum Apicomplexa also display short actin fibers that enables a rapid protein turnover and thus leads to negative imaging of actin fibers^31–33^. However, the structural and molecular relatedness of these intriguing actin molecules of Myxozoa to those of other parasitic species highlights the need for a better understanding of their roles by functional study. Here, we report KD of atypical actins in *S. molnari* cells. Following recently described protocols for parasite extraction^26^, we optimized and tested the systemic delivery of dsRNA into in vitro cultured parasite cells. We observed the phenotypic effect of silenced *ACT1* on parasite cell motility. Thus, this study highlights the successful utilization of RNAi in Myxozoa and opens new paths for gene function studies like mechanisms of drug resistance, host evasion tactics or developmental processes in these ancient and remarkable parasitic organisms.

## Results

### Efficient gene knockdown requires complete DNA removal

**Figure 1:**
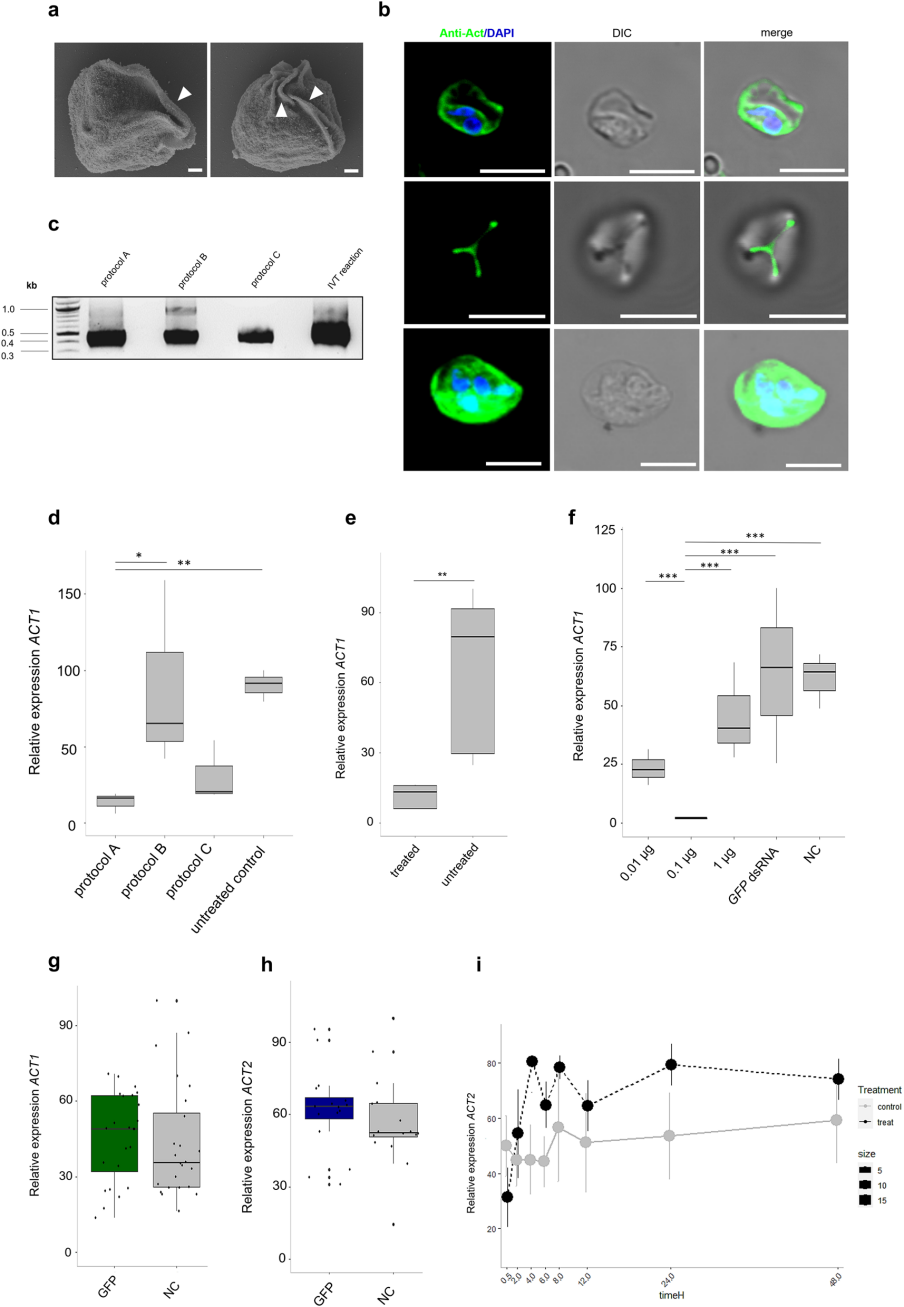
Optimization of dsRNA purification and testing the efficacy of the RNAi functional assay in *Sphaerospora molnari* blood stages. **(a)** Scan electron microscopy of *S. molnari* blood stages with typical membrane folds (indicated by white arrows). (**b**) ACT1 protein localization in *S. molnari* blood stages showing concentration in membrane folds (first and second line) and in the cytoplasm of primary cell (third line). ACT1 staining antibody (green) and nuclear staining with DAPI (blue), interference contrast image (DIC), *ACT1* and DAPI merged with the DIC. Scale bars—1 µm for (**a**) 5 µm for (**b**). (**c**) Purified *ACT1* dsRNA visualized by agarose gel electrophoresis (protocol A (lane 1): DNAse treatment followed by phenol/chloroform extraction and overnight ethanol precipitation, protocol B (lane 2): DNAseI treatment and ethanol precipitation, protocol C (lane 3): Manufactured protocol of dsRNA purification including digestion of shRNA and dsDNA contamination, in vitro transcription reaction (IVT reaction; lane 4): in vitro transcribed dsRNA from manufactured protocol. Original gel is presented in Supplementary Fig. 6. (**d**) Comparison of *ACT1* gene knockdown in tested protocols of dsRNA purification (A–C) described in (**c**) and negative (untreated) control after 24 h post incubation (n = 3). X-axis: protocols of dsRNA purification described in (**c**) and negative control *ACT1* gene expression in *S. molnari* cells (untreated control), Y-axis: relative gene expression values of *ACT1*. (**e**) Relative gene expression of ACT1 gene knockdown in *S. molnari* cells with purified dsRNA from protocol A (n = 5). X-axis: *ACT1* KD cells (treated) and negative control *ACT1* gene expression in *S. molnari* cells (untreated), Y-axis: relative gene expression values of *ACT1*. (**f**) dsRNA-concentration dependent relative gene expression of *ACT1* (n = 3)*.* X-axis: serial concentrations (µg/µl), Y-axis: relative gene expression values of *ACT1*. Housekeeping genes EF2 and GAPDH were used as the reference. (**g**) Relative gene expression of *ACT1* in *GFP* non-target control dsRNA and untreated negative control (NC) over 48 h of KD (n = 24). Y-axis: relative gene expression values of *ACT1*. Black circles represent jittered raw data. (**h**) Relative gene expression of *ACT2* on *GFP* control dsRNA and untreated negative control (NC) over 48 h of KD (n = 16). Y-axis: relative gene expression values of *ACT1*. Black circles represent jittered raw data. (**i**) Off-target effect analysis of relative gene expression of *ACT2* upon knockdown of *ACT1* (n = 3). X-axis: time points in hours (timeH), Y-axis: relative gene expression values of *ACT1*. In (**d–h**), housekeeping genes EF2 and GAPDH were used as the reference. Mean values relate to a dashed (treatment) or continuous (control) line over time. Error bars represent standard errors. In the boxplots, black horizontal lines represent the median, boxes represent 50% of the values, and upper and lower whiskers represent values > 75th and < 25th percentiles. Significant differences between groups are indicated in the figures with an asterisk, following ∗p < 0.05, ∗∗p < 0.01, ∗∗∗p < 0.001.

We investigated various approaches to test and improve gene KD efficiency in *S. molnari* cells. Given the formerly identified atypical beta-actins of *S*. *molnari*^28^, we selected two homologs (*ACT1* and *ACT2,* Supplementary Fig. 1), with emphasis on the unusual Actin1 protein (*ACT1*), localized in the primary cell cytoplasm and membrane folds of *S*. *molnari* blood stages (Fig. 1a, b). First, we produced dsRNAs targeting *ACT1* (Supplementary Fig. 1) using three different protocols (protocols A–C) and examined their purity on agarose gel (Fig. 1c). Apart from one approach showing the presence of incomplete removal of DNA template displayed by an upper band (Fig. 1c, protocol B), we obtained single band size purified dsRNA (Fig. 1c). We then estimated maximum silencing efficiency of *ACT1* using RT-qPCR and calculated the percentage of decrease (Fig. 1d), reaching 85%, by applying DNAse I treatment followed by phenol/chloroform extraction and ethanol precipitation (Fig. 1c, protocol A), compared to untreated control (Fig. 1d, LM, est = 2.522, SE = 0.351, p < 0.001). Omitting the phenol/chloroform step (Fig. 1c, protocol B) resulted in a slightly lower silencing efficiency of *ACT1* (2% less compared to protocol A; Fig. 1d), without significant difference (LM, est = 0.173, SE = 0.446, p = 0.979). In contrast, the manufacturer’s purification protocol of the commercial dsRNA synthesis kit showed silencing efficiency comparable to protocol A (66%), however, the low yield of purified dsRNAs resulted in inconsistencies in gene silencing of both *ACT1* and *ACT2* in follow-on experiments (Supplementary Fig. 2a, b). Additionally, we tested unpurified in vitro transcribed dsRNA (Fig. 1c, Supplementary Fig. 1) in RNAi assays but did not observe consistent gene KD for either of the actin homologs (*ACT1* and *ACT2*) across experiments, predicting potentially unreliable outcomes in future experiments. The consistency of effective gene KD observed in dsRNA produced by protocol A was further confirmed by five independent biological replicates, showing low variation of downregulation of gene expression across experiments (Fig. 1e, LM, est = 1.657, SE = 0.371, p = 0.002).

### RNAi efficiency in *S. molnari* shows dose-dependent uptake of dsRNA

To determine the optimal concentration of dsRNA for efficient gene KD, we tested three different concentrations of *ACT1* dsRNA ranging from 0.01 to 1 µg/µL (Fig. 1f), including one untreated control (NC) and a non-target control utilizing a gene non-existent in Myxozoa (e.g., green fluorescent protein (GFP), hereafter *GFP* dsRNA). RT-qPCR analysis of mRNA levels of *ACT1* revealed that the most effective gene silencing, with a reduction of gene expression of 97%, was achieved at a concentration of 0.1 µg/µL (Fig. 1f), showing significant differences to all other groups (LM, est = −3.075 to 3.434, SE = 0.342, p < 0.001). The use of a tenfold higher concentration (1 µg/µL) resulted in only a 27% reduction, while the lower concentration (0.01 µg/µL) showed 63% reduction compared to untreated control samples (Fig. 1f), although none of these differences were significant (LM, posthoc est = −0.635 to 0.995, SE = 0.342, p > 0.05). We also verified the efficiency and specificity of our RNAi assay using non-target *GFP* dsRNA while measuring gene expression of *ACT1* and *ACT2* prior to silencing our target genes. In both actin genes, there was no overall unintended pattern of downregulation in gene expression compared to untreated controls, over a 48-h period of incubation (Fig. 1g, h). Neither the relative expression of *ACT1* (Fig. 1g) nor *ACT2* (Fig. 1h) differed between the *GFP* group and the control (LM, *ACT1*: est = −0.075, SE = 0.137, p = 0.586; *ACT2*: est = −0.108, SE = 0.131, p = 0.415). Next, we inspected the off-target effect by measuring gene expression of the close gene ortholog *ACT2* by KD of *ACT1* (Fig. 1i). While *ACT1* was silenced, we did not observe differences in the relative expression of *ACT2* between treatments or over time (LM, treatment: est = −0.207, SE = 0.163, p = 0.212; time: est = −0.005, SE = 0.008, p = 0.530).

### dsRNA uptake and soaking strategy in *S. molnari* blood stages

**Figure 2:**
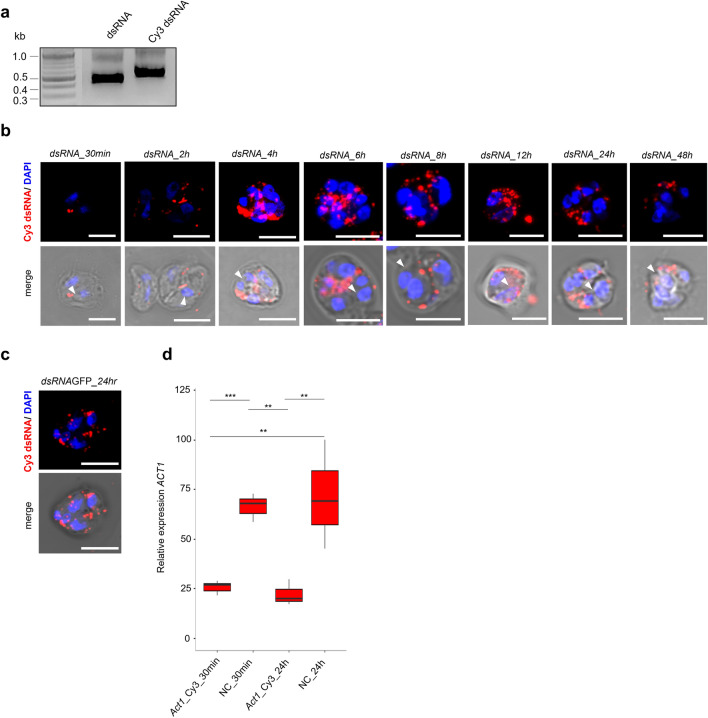
Cellular uptake of dsRNA in *S. molnari* cells. **(a)** Negative color agarose gel of purified dsRNA (dsRNA) and Cy3-labeled dsRNA (Cy3 dsRNA). Ladder indicates band sizes in kilobases (kb). Original gel is presented in Supplementary Fig. 7. (**b**) Cellular uptake of Cy3 dsRNA by *S. molnari* blood stages over time (30 min to 48 h) in KD assay, Cy3 (red, dsRNA) combined with nuclear DAPI staining (blue). Secondary cells are indicated by arrows. (**c**) Cy3-labeled *GFP* dsRNA in *S. molnari* blood stage after 24 h post incubation. (**d**) Relative gene expression of *ACT1* by *S. molnari* soaked with Cy3-labeled *ACT1* dsRNA, after 30 min and 24 h of incubation (n = 3). Bars indicate gene expression relative to untreated control samples (NC). X-axis: Cy3-labeled samples (Act1_Cy3) and negative controls (NC). Y-axis: relative gene expression values of *ACT1*. Housekeeping genes EF2 and GAPDH were used as the reference. In the boxplots, black horizontal lines represent the median, boxes represent 50% of the values, and upper and lower whiskers represent values > 75th and < 25th percentiles. Significant differences between groups are indicated in the figures with an asterisk, following ∗p < 0.05, ∗∗p < 0.01, ∗∗∗p < 0.001. Scale bars—5 µm.

To prove that dsRNA was successfully taken up by *S*. *molnari* cells using the soaking approach, we utilized Cy3-labeled dsRNA of *ACT1*. Prior to incubation, we examined labeled dsRNA on an agarose gel to ensure the correct fluorescent labeling procedure (Fig. 2a). While incubating labeled dsRNA with *S*. *molnari* cells, we detected an increasing accumulation of perinuclear foci in the cytoplasm of primary and secondary cells, over time, using confocal microscopy (Fig. 2b). Interestingly, the internalized dsRNA appeared within the primary cell cytoplasm after 30 min post-incubation (Fig. 2b). Additionally, we performed a soaking assay with non-target control encoding *GFP* dsRNA and found similar internalization and distribution of labeled dsRNA within *S. molnari* cells after 24 h (Fig. 2c). We simultaneously analyzed gene expression of *ACT1* while using Cy3-labeled dsRNA, which resulted in effective KD of the target *ACT1* gene after 30 min and 24 h compared to untreated controls (Fig. 2d, LM, posthoc 30 min: est = 0.954, SE = 0.214, p = 0.009, and 24 h: est = 1.142, SE = 0.214, p = 0.003).

### RNAi functional assay in *S. molnari* cells

**Figure 3:**
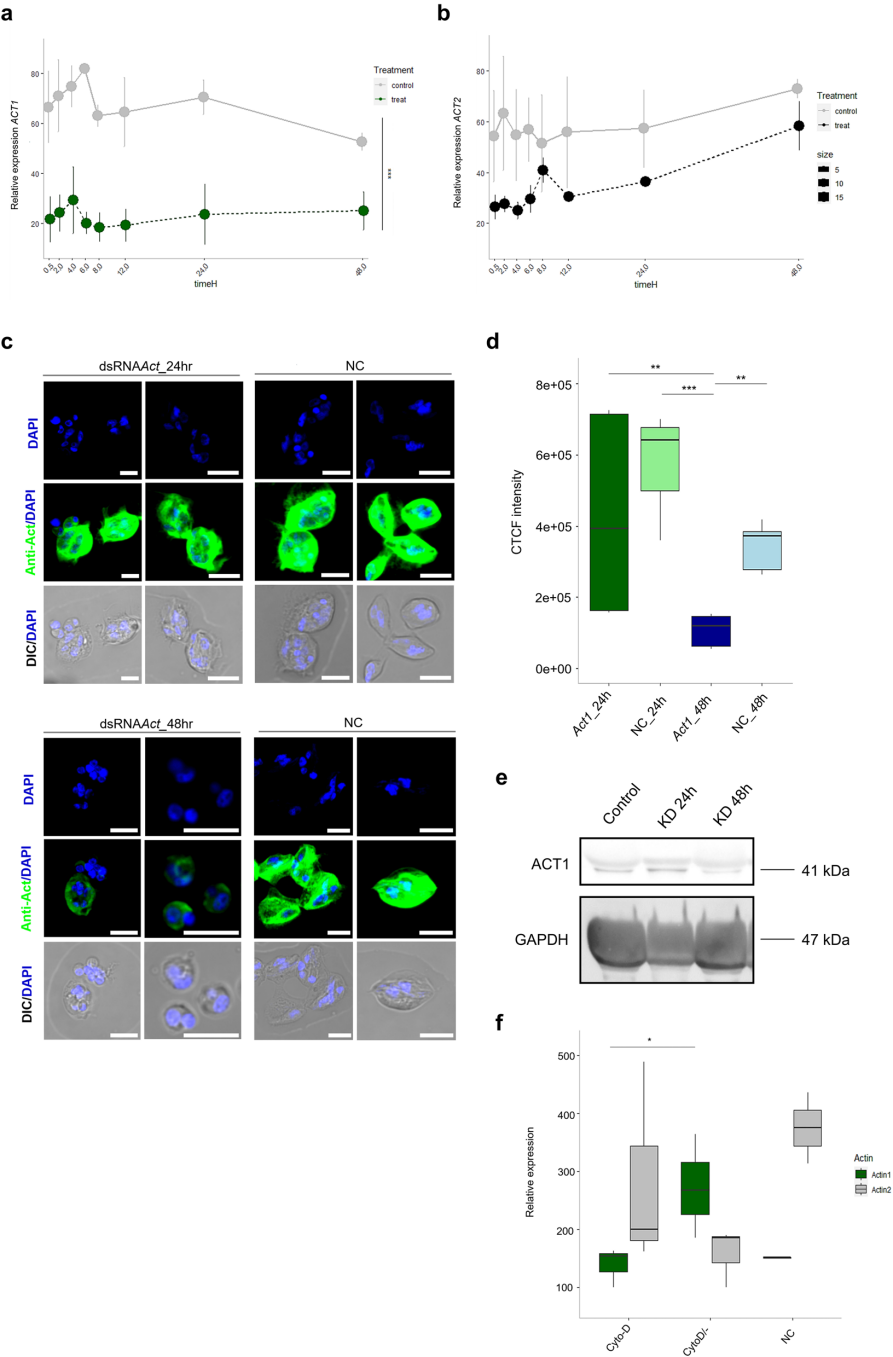
Knockdown assays in *S*. *molnari* blood stages and expression of actins in cells during actin polymerisation inhibition. **(a)** Relative gene expression of *ACT1* KD (green dots) compared to negative control (grey dots) in *S. molnari* over 48 h of gene silencing (n = 3). (**b**) Relative gene expression of *ACT2* KD (black dots) compared to negative control (grey dots) in *S. molnari* cells over 48 h (n = 3). In (**a,b**), mean values are connected with a dashed (treatment) or continuous (control) line over time. Error bars represent standard errors. **c** Immunolabeling of *S. molnari* blood stages stained for ACT1 (green) showing slight decrease of protein expression in KD *S. molnari* cells after 24 h (*ACT1* KD 24 h) and strong decrease after 48 h (*ACT1* KD 48 h) compared to control cells (NC). Nuclei are stained with DAPI (blue) and merged with differential contrast images (DIC). Scale bars—5 µm.** (d)** Corrected total fluorescent intensity (CTCF) in *S*. *molnari* cells after 24 h and 48 h compared to control cells (NC) (n = 5). (**e**) Western blot showing protein quantities of ACT1 and GAPDH (housekeeping gene) after KD of *ACT1* in *S*. *molnari* cells after 24 h (KD 24 h), and 48 h (KD 48 h), displaying reduced protein level of *ACT1* at 48 h in the RNAi group vs the Control. Original blots are presented in Supplementary Fig. 8. (**f**) Relative gene expression of *ACT1* (green box plots), *ACT2* (grey box plots) and negative control (NC) in *S. molnari* cells exposed to cytochalasin D (Cyto-D) (n = 3). X-axis: cytochalasin D-treated cells (Cyto-D), washed Cyto-D cells (Cyto-D/−) and negative control (NC). Y-axis: relative gene expression values of *ACT1* and *ACT2*. Housekeeping genes EF2 and GAPDH were used as the reference. In the boxplots, black horizontal lines represent the median, boxes represent 50% of the values, and upper and lower whiskers represent values > 75th and < 25th percentiles. Significant differences between groups are indicated in the figures with an asterisk, following ∗p < 0.05, ∗∗p < 0.01, ∗∗∗p < 0.001.

We conducted a functional RNA interference (RNAi) assay to investigate the effect of gene KD on *ACT1* and *ACT2* genes in *S. molnari* blood stages. Our analysis revealed a significant reduction in mRNA expression levels of both actin genes after 30 min, which persisted for up to 48 h (Fig. 3a, b). The relative expression of both, *ACT1* and *ACT2* was lower in the treatment than in control (LM, *ACT1* treatment: est = 1.093, SE = 0.190, p < 0.,001; *ACT2* treatment: est = 0.564, SE = 0.162, p = 0.001), showing no significant differences over time for *ACT1* (Fig. 3a LM, time: est = −0.006, SE = 0.007, p = 0.414) and a slight increase over time in both groups for *ACT2* (Fig. 3b, LM, time: est = 0.016, SE = 0.006, p = 0.009). We also examined *S*. *molnari* cells under the confocal microscope and observed no difference in fluorescence after 24 h (Fig. 3c). However, a lower fluorescent labeling signal was detected after 48 h of KD (Fig. 3c). Subsequently, we performed fluorescence intensity quantification of KD in *S*. *molnari* (Fig. 3d). Corrected total cell fluorescence (CTCF) intensity analysis (Fig. 3d) revealed a significant decrease of *ACT1* after 48 h compared to 24 h and to controls at 24 h and 48 h (LM, posthoc, est = −1.270 to 1.742, SE = 0.305, p = 0.004- < 0.001). The comparison of the fluorescence intensity with protein concentration in the cells using western blotting showed a considerable reduction in protein expression levels after 48 h of KD (Fig. 3e). However, no substantial decrease of protein was detected at 24 h compared to control in our RNAi assay (Fig. 3e).

### Cytochalasin D removal induces recovery of gene expression of atypical actins

To understand the expression dynamics of target actins upon inhibition and reversibility of actin polymerization, we incubated *S*. *molnari* blood stages with cytochalasin D (Cyto-D) for 24 h. Overall, the removal of Cyto-D from cell culture resulted in the recovery of gene expression for both actin genes (Fig. 3f). After 24 h of incubation with Cyto-D, relative gene expression levels of *ACT1* and *ACT2* did not show significant differences between cytochalasin D-treated cells (Cyto-D) and the untreated negative control (LM, *ACT1* est = 0.109, SE = 0.249, p = 0.680; *ACT2* est = 0.385, SE = 0.409, p = 0.389). However, following the removal of Cyto-D after 30 min of incubation, gene expression of *ACT1* increased compared to the Cyto-D treated cells (LM, est = 0.657, SE = 0.222, p = 0.03), suggesting a recovery of gene expression for *ACT1*. This trend was not the case for *ACT2*, as gene expression for *ACT2* did not show any changes after the removal of Cyto-D (LM, CytoD/−: est = −0.500, SE = 0.366, p = 0.229).

### Silencing of actin results in disintegration of actin cytoskeleton of *S. molnari* primary cells

**Figure 4:**
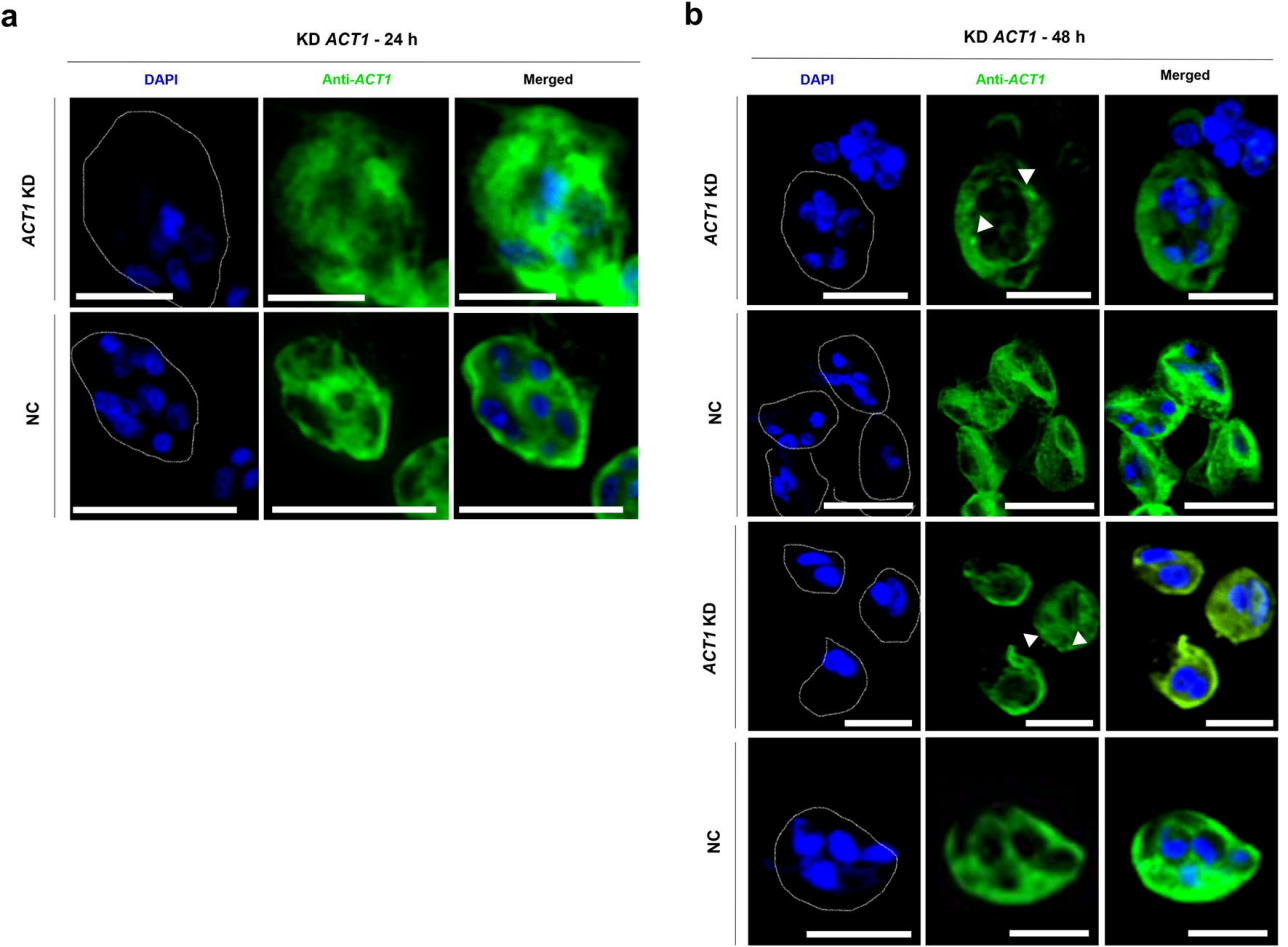
Actin knockdown in *S. molnari* blood stages shows disintegration of cytoskeleton in cytoplasm of the primary cells. **(a,b)** Immunolabeling of *S. molnari* blood stages stained for ACT1 (green) and DAPI-stained nuclei (blue) showing lack of actin network in cytoplasm of primary cells and appearance of actin patches (indicated by white arrows) after 24 h (**a**) and 48 h (**b**) of *ACT1* KD compared to untreated controls (NC). Scale bars—5 µm.

To investigate the silencing of *ACT1* on the cytoskeleton of *S*. *molnari* blood stages, we visualized *ACT1* in parasite cells post RNAi. We observed distinct changes in the cytoskeleton of the primary cell cytoplasm of blood stages (Fig. 4a, b). *ACT1* KD cells showed a disintegrated actin network in the primary cell cytoplasm compared to untreated controls after 24 h (Fig. 4a). The protein signal of *ACT1* was largely absent from the cytoplasm of primary cells after 48 h (Fig. 4b). Moreover, we detected the appearance of actin patches at the cell periphery in *ACT1* KD cells after 48 h (Fig. 4b). Control parasites displayed actin-rich areas in either the cytoplasm or the membrane of the primary cells after 24 h and 48 h (Fig. 4a, b).

### Gene knockdown altered cell rotational movement of *S. molnari*

We investigated the effect of dsRNA-mediated KD of *ACT1* and *ACT2* on the phenotype of *S. molnari* blood stages. Overall, there was no alternation observed in the membrane folds emergence upon KD of both *ACT1* and *ACT2* on the cell surface of *S. molnari* blood stages. We observed loss of axial motility with continuous forming of membrane folds in *ACT1* KD cells (Supplementary video 1) compared to untreated control (Supplementary video 2) and *GFP* dsRNA-treated cells at 48 h (Supplementary video 3). These findings were consistent with the reduced fluorescence intensity observed after 48 h (Fig. 3d), while neither motility nor fluorescence intensity was altered after only 24 h of *ACT1* KD (Supplementary video 4) when compared to the *GFP* dsRNA group (Supplementary video 5) and the untreated control (Supplementary video 6). Furthermore, western blot analysis revealed a drop in protein expression at 48 h but without a complete lack of protein expression in *S. molnari* (Fig. 3e). Conversely, KD of *ACT1* did not result in any significant changes in cell morphology compared to control and non-target-treated cells. Silencing of *ACT2* did not alter cell motility of *S*. *molnari* blood stages after 24 h or 48 h of KD (Supplementary videos 7 and 8).

## Discussion

In the present study, we performed the RNAi-mediated gene knockdown (KD) of two atypical actins, *ACT1* and *ACT2*, within the causative agent of common carp gill sphaerosporosis, *Sphaerospora molnari*. Our study exemplifies gene silencing confined exclusively to purified cultured stages of the parasite. A previous study^20^ employed small interfering RNA (siRNA) targeting a myxozoan serine protease, delivered through soaking an infected oligochaete host. This approach demonstrated sustained knockdown of myxozoan-infected oligochates collected over the 3 months period, with the most pronounced gene silencing observed at the 24-h mark. The transient nature of RNAi, typically lasting only for a few days^4,34^, requires the continuous presence of either siRNA or double-stranded RNA (dsRNA), consistent with our findings of sustained knockdown for a duration of 48 h.

To ensure RNA inhibition in all cells of the unique cell-in-cell structure of myxozoans, we used a simple soaking strategy of dsRNA into myxozoan multicellular cells, rather than applying short hairpin RNA molecules (siRNA) via electroporation. The utilization of the electroporation delivery system poses challenges for Myxozoa due to the cell-in-cell structure observed in myxozoan proliferative stages, impeding the successful integration of shRNAs into all cells within these stages. We optimized the purification and dosage of dsRNA in the parasite cell culture. Owing to the perplexing inconsistencies in RNA yield and gene KD results observed with commercially produced dsRNA, we tested alternative approaches for dsRNA purification. Hence, we presume that the low yield of dsRNA obtained is likely a result of incomplete elution, attributed to the limited presence of chaotropic agents and cations in the buffers of commercial kits, as reported^35^. In comparison to other studies using dsRNA precipitation^36^ and phenol/chloroform extraction^37^, we amended the stable production of pure and functional dsRNA. As demonstrated, we omitted the use of RNAse A in our approach based on previous results that showed the susceptibility of dsRNA to RNAse A digestion^38^. Our results support previous applications of the cost-effective soaking approach for introducing dsRNA into cells^4,7,39^. Regardless of delivery methods for RNAi, rapid cellular uptake of dsRNA into cells has been reported^40–42^. Similarly, *S. molnari* cells displayed the presence of intracellular dsRNA within 30 min of incubation, allowing for further systemic spread within the complex cell-in-cell stage structures defined for myxozoans^43^. However, the exact mechanism of cell entry of dsRNA in Myxozoa remains unclear. We have not yet identified the transmembrane protein SID-1 in the transcriptome of *S. molnari* (PRJNA522909)^44^ that allows passive cellular uptake as an entry transporter of dsRNA, described in model nematode *Caenorhabditis elegans*^45,46^. Conversely, as the essential role of SID for systemic effect and cell-to-cell transport of dsRNA, the absence of this evolutionarily conserved homolog in Myxozoa suggests yet unknown machinery of systemic spread of RNAi in myxozoan multicellular proliferative stages. Nonetheless, the lack of this protein can be sustained by scavenger receptors, mediating the uptake mechanism^47,48^. Alternatively, the cellular uptake might be triggered by endocytosis^49^. In our study, we found that continuous exposure to dsRNA is necessary to achieve long-term effective gene KD. However, long-term in vitro culture for the temporary gene silencing in culture is quintessential to mimic in vivo conditions^50^. Our experiments showed that maximum gene silencing effect and cell viability were observed up to 48 h, but prolonged co-culture with dsRNA for 62 h resulted in inconsistent and less effective knockdown and rapid cell death (Supplementary Fig. 3). This was likely a result of parasites dying in the in vitro culture. Longer-term survival of *S*. *molnari *in vitro would be an essential requirement to study the longevity of the KD effect in vitro and in vivo within the intermediate host but has not presently been achieved. We empirically determined an optimal concentration of dsRNA to trigger efficient gene KD that shows a maximum gene silencing effect at a lower concentration of dsRNA rather than higher. Such concentration-effect has been also observed using RNAi in other organisms^51^. To some extent, higher doses of dsRNA show more robust gene silencing^52^. By contrast, our results demonstrate low efficiency of gene knockdown while applying a high concentration of dsRNA, also reported in studies primarily using insect model species^53^. However, inefficient RNAi employing a high dose of dsRNA is hypothesized with increasing RNAse activity that instigates degradation of dsRNA^54^. In contrast, siRNA-based gene silencing displays opposite trends and increasing efficiency at higher doses^55–57^. Further characterizations showed that several mechanisms and factors influence efficiency mostly in insect models^58–60^. The factors that affect RNAi efficacy in *S*. *molnari* blood stages with higher concentrations are yet unknown. In this study, we used GFP as a non-target exogenous control based on the absence of this gene in Myxozoa genomic data, although this gene is endogenously present in free-living Cnidaria^61^ as well as other GFP-like homologs across Metazoa^62^. While several reports regarding off-target or indirect effects of GFP on gene expression in RNAi studies exist^63–65^, we anticipated that using GFP as non-target control for RNAi-based studies in our model species *S*. *molnari* can be employed. However, deeper profiling of differential gene expression in knockdown targeting GFP could explore any unintended non-target effects on a broad scale. Additionally, unlike the previous study using one predesigned siRNA control in Myxozoa^20^, we matched another negative control to each silenced target to monitor the expressional profiles of the targeted actins and validate experimental conditions during RNAi experiments.

We demonstrated here for the first-time knockdown targeting atypical beta-actin orthologs based on their suspected role in an unconventional type of cell motility of *S. molnari* blood stages, previously reported by^28^. Knockdown of *ACT1* showed decreased fluorescence signal that supports downregulation of the gene expression in *S*. *molnari* blood stages. However, in comparison with siRNA-based beta-actin gene silencing in human fibroblasts^66^, a total absence of the fluorescent signal was not observed in our experiments. Overall, *ACT1* knockdown altered cell motility of *S*. *molnari* blood stages. However, any deleterious phenotypic effects, including degradation of overall cell structure or rapid cell death, were not observed. This result is in striking contrast with altered tissue morphology of beta-actin knockdown in evolutionarily related cnidarian sea anemone *Aiptasia pallida*^67^. In our model *S*. *molnari*, the reduced expression of *ACT1* during KD led to an apparent lack of rotational movement of the cells under culture conditions, together with subcellular localization showing a lower signal of the protein within the primary cell membrane after 48 h of KD. We note that, despite successful *ACT1* KD during the assay, the cells retained the ability of MFIT motility, suggesting the role of actomyosin contractility in *S*. *molnari* motility^28^ and incomplete ablation of *ACT1* gene expression. This may constrain the application of RNAi in gene function studies^68,69^. However, the exact machinery triggering the rotational movements of *S*. *molnari* around the axis is not completely understood. It is likely that cell polarity and microtubule dynamics play a key role in rotational motion^70^. Immunolabeling analysis of cells with silenced *ACT1* revealed disintegration of the actin cytoskeleton in primary cell cytoplasm accompanied by the appearance of actin patches^71^. This less extensive actin network was also observed in knockdown of actin genes in gene-silencing studies in other organisms^50,72^. Nevertheless, *S*. *molnari* cells were still viable and unimpaired. This might indicate the dispensable role of actins as observed by complete gene deletion via knockout assays in mice models^73^. Therefore, it is tempting to note that changes in the cytoskeleton, notably actin patches can be coupled with changes related to the cell recovery after actin depolymerization^74^. To better understand, a future assay of cell proliferation and apoptosis can explore cytoskeletal changes upon gene silencing in *S*. *molnari*. Overall, suppressing the gene expression of ACT2, whose cellular distribution in *S*. *molnari* is still unknown, did not alter cell motility. This is coherent with previous observations reporting no effect on the cell speed upon knockdown of beta-cytoplasmic actin^75^. Disruption of actin polymerization with cytochalasin D (Cyto-D) suggests that actomyosin contractility is an important factor for motile cells^28,76,77^. As previous evidence of Cyto-D removal profoundly affects the return of actin gene expression to basal levels^78^, both targeted actin orthologs display similar gene recovery. Conversely, Cyto-D induces actin mRNA expression with protein synthesis targeting the regulatory region of beta-actin promoters^78^. Intriguingly, *S*. *molnari* cells treated with Cyto-D exhibit elevated mRNA levels of the *ACT2* ortholog. This suggests that pharmacological inhibition of actins induces the synthesis of other actin genes while altering cytoskeletal organization^79^. It is tempting to speculate whether *ACT2* is hence involved in cell division^80,81^ or contributes to other non-redundant biological roles^82^. Indeed, KD of both actin isoforms did not result in the reduction or loss of membrane folds creation in motile *S*. *molnari* blood stages. This might indicate that the membrane folds on the surface of the *S. molnari* cells could be driven by other factors, such as tyrosine kinases activating actin remodeling and, thus, the formation of these membrane extensions^83^.

Currently, the major obstacle for further RNAi research in myxozoans lies in the lack of stable long-term in vitro-based culture of myxozoan parasite stages that would enable the study of the developmental processes upon gene editing. In our work, the parasite viability limited us to monitor the longevity of RNAi knockdown. Despite parasite cell proliferation and short-term viability in cultures reported^23^, the proliferation of the parasitic stages and plasmodium-spore transition under cultured conditions need further optimization. Expanding the range of species and adapting our optimized RNAi technique could benefit future studies. Despite the lack of knowledge in driving components beyond the motility in *S*. *molnari* blood stages, the RNAi approach in our in vitro system allows functional studies of other genes. Ultimately, the transgenesis in Myxozoa, encompassing gene knockout and other genome-editing methodologies, represents a significant advancement in the exploration of gene function within these economically significant and evolutionarily derived parasites.

## Methods

### Ethical statement

All experimental protocols were approved by the Resort Professional Commission of the CAS for Approval of Projects of Experiments on Animals. Prior to the collection of *S. molnari* blood stages, the fish specimens were subjected to euthanasia utilizing clove oil. The manipulation and sampling protocols were executed with a consistent approach and in strict adherence to the provisions of the Czech legislation governing the welfare of animals, as set forth in the Protection of Animals Against Cruelty Act No. 246/1992. All procedures were authorized by the Czech Ministry of Agriculture. The study is reported in accordance with ARRIVE guidelines (https://arriveguidelines.org). The parasite proliferative stages were isolated from the full blood (0.5–2 mL) collected from intraperitoneally injected pathogen-free (SPF) carps (total length 12.5–16 cm) using ion-exchange chromatography as outlined by^26^.

### Cell culture

Isolated parasite blood stages were seeded into 96-well plates at a density of 200,000 cells per well and subsequently cultured in RPMI 1640 medium (Gibco, Life Technologies) at 26 °C with 5% CO_2_. The medium was fortified with 20% heat-inactivated fetal bovine serum (Biosera Europe, France), 1 mL of GlutaMAX™ (ThemoFisher, USA), carp red blood cell lysate equalling 1:10 parasite cells: host cells (host cells lysed in water), and 0.008 mL of antibiotics/antimycotics (10,000 U/mL, Sigma-Aldrich, USA).

### In vitro transcription

To utilize RNAi in *S*. *molnari*, we employed dsRNA regarding higher efficacy and cost-effective application compared to siRNAs (reviewed in^84^). We selected in vitro transcription of dsRNA regarding rational experimental design and the potential off-target effect of dsRNAs according to the parameters reported by^60^. The cDNA templates of ACT1 and ACT2^28^ were amplified using standard PCR and gene-specific primers bearing 5′ end tags of the T7 TAATACGACTCACTATAGGGAGA promoter sequence (Invitrogen, USA) (Supplementary Table 1). The amplified PCR products of optimal length (~ 500 bp) were subjected to electrophoresis on 1% agarose gel, extracted, and purified using a Gel/PCR DNA Fragments Extraction Kit (Geneaid Biotech Ltd, Taiwan). Subsequently, in vitro transcription was conducted by incubating 1 µg of cDNA template at 37 °C for 16 h using the MEGAscript™ T7 Transcription Kit (Invitrogen, USA), following the manufacturer's protocol. We chose green fluorescence protein (GFP) encoding sequence as a non-target control dsRNA since is absent in Myxozoa, which also supports no hits in the transcriptome search of *Sphaerospora molnari* (PRJNA522909)^44^. Plasmid pkpl1, containing the GFP sequence insert, was kindly provided by Dr. O. Hajdušek from the laboratory of J. Perner, Institute of Parasitology, Biological Centre of the CAS, Czech Republic. One microgram of the plasmid was linearized, and the GFP insert was excised from the backbone using XbaI/AbaI restriction enzymes (Invitrogen, USA), followed by electrophoresis on a 1% agarose gel (Supplementary Fig. 4). The cleaved GFP insert was extracted using a Gel/PCR DNA Fragments Extraction Kit (Geneaid Biotech Ltd, Taiwan). The GFP T7-PCR template was amplified with T7-specific primers (Supplementary Table 1), and the resulting in vitro transcribed *GFP* dsRNA was tested for off-target effects on target gene expression by RT-qPCR. Thereafter, a custom synthetic non-target GFP dsRNA was acquired from Creative Biogene (New York, USA) for use in further RNAi assays.

### Optimization of dsRNA purification

Despite the setback with inefficient gene silencing of in vitro transcribed and purified dsRNAs via conventional manufacturing procedure, we troubleshot and optimized the purification of dsRNA as the crucial step for the successful induction of RNAi in our target model species. The dsRNA encoding *ACT1* was purified to remove any residual DNA template from the in vitro transcription reaction. Three different protocols (protocols A–C) were tested, involving either inclusion (protocol A) or exclusion (protocol B) of DNAse I treatment, phenol/chloroform extraction (protocol B), and overnight ethanol precipitation (protocol C), with three replicates each. In protocol A, the dsRNA was treated with DNAse I for 1 h at 37 °C, followed by increasing the volume with RNAse-free H_2_O to 100 µL, and extraction with phenol/chloroform isoamyl alcohol (25:24:1) at room temperature. The upper phase was collected in each step. Next, the dsRNA was incubated with 2.5 equivalent volumes of RT 100% EtOH and 3 M NaOAc, overnight at −20 °C. Precipitated dsRNA was centrifuged at 15,000*g* for 30 min, at 4 °C, and 5 min at top speed after being washed with 70% cold EtOH. The purified dsRNA was air dried, resuspended in the original volume of 20 µL, verified on agarose gel, and the final yield was measured on the Nanodrop. For Protocol B, we omitted the phenol/chloroform extraction step, while the remainder of the purification procedure was like protocol A. In protocol C, purification of in vitro transcribed dsRNA was performed using the MEGAscript™ T7 Transcription Kit (Invitrogen, USA) according to the manufacturer’s instructions. Subsequently, parasite stages were soaked with dsRNA, and gene KD efficiency was evaluated by RT-PCR of the gene target after 24 h, compared to the untreated control sample. Relative change in expression values was then calculated as a percentage change [relative expression change = ΔV/V_1_ × 100%: ΔV defines the difference between the final average expression value of KD (V_2_) and negative control expression value (V_1_)]. To verify the consistency of stable gene silencing, five biological replicates were used comparing the differences in *ACT1* gene expression in treated (protocol A) and untreated samples.

### Fluorescent labeling of dsRNA

In search for optimal and effective delivery of dsRNA into the daughter cells of the cell-in-cell myxozoan stages, we employed fluorescent labeling using the Silencer™ siRNA Labeling Kit with Cy™3 dye (Invitrogen, USA) to label dsRNA of *ACT1* and non-target control *GFP* (hereafter Cy3-labeled dsRNA). The labeling process involved dsRNA incubation with 10× Labeling Buffer and Cy3™ dye at 37 °C for 1 h. Next, we added 5 M NaCl (0.1 × total volume) and 100% pre-cooled EtOH (2.5 × total volume), mixed the solution thoroughly, and incubated it at −20 °C for 30 min. The precipitated dsRNA was then centrifuged at top-speed (12,100*g*) at 4 °C for 20 min, and the resulting pellet was washed with 70% EtOH, followed by additional top-speed centrifugation and air drying of the pellet. Finally, the labeled dsRNA was resuspended in the original volume of RNAse-free H_2_O. We verified the presence of the labeled dsRNA by electrophoretic assay on 1% TBE agarose gel and compared it to unlabeled dsRNA after 30 min and 24 h (n = 3, Fig. 2D). Simultaneously, *S*. *molnari* cells were left to soak Cy3-labeled dsRNA for 48 h and checked under Olympus FluoView FV3000 confocal laser scanning microscope equipped with FluoView FV31S-SW software (v. 2.6) with eight sampling time points (30 min, 2, 4, 6, 8, 12, 24, 48 h).

### RNAi in vitro experiments

Given the successful preliminary gene silencing, we performed RNAi assays using our optimized workflow. We targeted both actins for 48 h with eight sampling time points (30 min, 2, 4, 6, 8, 12, 24, 48 h). All experiments were performed in at least three biological and technical replicates in the total amount of 100 µL. Each experiment consisted of cells undergoing knockdown supplemented by negative control of cultured cells to monitor the expressional profile of targeted actins and one additional control using GFP dsRNA to elucidate the non-target effect during knockdown over the gene silencing duration. The cell motility was monitored in real-time timelapse videos (29.97 frames per second) using an inverted light microscope Olympus IX70. The cells were harvested and stored in an ICL fixation buffer (Qiagen, Germany) at −20 °C for RNA extraction and RT-qPCR analysis or subjected to immunolabelling or western blotting procedures, described below.

### Live cell imaging

To evaluate the effect of the knockdown of both actins on *Sphaerospora molnari* blood stage motility, we performed live cell imaging. We recorded *S*. *molnari* cells from RNAi assays with an inverted microscope Olympus IX70 and recorded on Canon 1300D camera. The 96-well-plated cells were imaged at 29,97 frames per second (in total 60 s) using a 40× objective lens. Recorded videos were then post-edited on the Fiji platform using ImageJ^85^.

### RNAi efficiency and non/off-target effect validation

To deliver sufficient dsRNA into the cells to achieve successful RNAi, different concentrations of *ACT1* dsRNA (final concentrations ranging between 0.01 and 1 µg/µL) were used for soaking the parasites stages and allowing dsRNA uptake. Differences in the relative expression of *ACT1* were tested on *GFP* dsRNA and untreated control (n = 3) to estimate the effectiveness of gene silencing at a particular concentration of dsRNA. For non-target control testing, we performed the RNAi assay with GFP dsRNA at a concentration of 1 µg/µL and incubated *S*. *molnari* cells for a total of 48 h. Cells were collected at eight sampling time points (30 min, 2, 4, 6, 8, 12, 24, 48 h) and subjected to RT-qPCR analysis targeting gene expression compared to the untreated control (n = 3). As reported previously^28^, *ACT1* and *ACT2* display moderate sequence similarity (around 70%). Thus, apart from analyzing the inhibitory effect on *ACT1*, we also performed an off-target effect RT-qPCR assay for *ACT2*.

### Immunolabelling

The question of whether our gene silencing of ACT1, previously described by^28^, has any effect on the actin cytoskeleton, we subjected *S*. *molnari* cells to confocal microscopy. For ACT1 staining, isolated parasite blood stages were first seeded on Superfrost Plus slides using a Cytospin (centrifugation at 800*g* for 5 min). The cells were subsequently fixed with IC fixation buffer containing 4% paraformaldehyde (Invitrogen, USA), for 30 min at room temperature. After fixation, the cells were washed three times with PBS and blocked at room temperature for 1 h in a solution consisting of 5% (w/v) skimmed milk in 0.3% Tween20 in PBS (= PBST). Next, the cells were incubated overnight with an anti-*S. molnari* actin 1 primary antibody (polyclonal, rabbit) at a 1:500 dilution^28^. After three washes with PBST, the cells were stained with goat anti-rabbit IgG-Alexa Fluor Plus 647 secondary antibody and DAPI for nuclear staining at dilutions of 1:10,000. Finally, the samples were mounted using Fluoroshield Mounting Medium (Searle Diagnostic Gurr Products, United Kingdom). The slides with the stained parasite cells were imaged with an Olympus FluoView FV3000 confocal laser scanning microscope equipped with FluoView FV31S-SW software (v. 2.6). Images were then deconvolved using DeconvolutionLab2^86^ implemented in the Fiji platform of the ImageJ software^85^. Labeled *ACT1* dsRNA and GFP uptake was verified via confocal microscopy at different time points from 30 min to 48 h. For each time point, cells were washed with PBS and cytospun on SuperFrost slides. The cells were then permeabilized in PBST and stained with DAPI after fixation in IC fixation buffer. The slides containing the parasite cells were imaged using confocal microscopy (see above). The fluorescent intensity was quantified by measuring the area-integrated intensity of cells and background in Z-stacks confocal images of cells (n = 10) across three independent biological replicates, using ImageJ. The corrected total cell fluorescence (CTCF) was calculated using GraphPrism (v. 6) with the formula: Integrated Density – (Area of selected cell × Mean fluorescence of background readings).

### qRT-PCR

As a reference validation for all RNAi assays, we monitored the gene expression profile of both targeted actins to check gene silencing efficiency and off-target and non-target effects. Cells that were subjected to RNAi in vitro assays were centrifuged at 800*g* for 10 min and fixed using RLT lysis buffer (Qiagen, Germany). The fixed cells were then stored at −20 °C until further use. Total RNA was extracted using RNeasy Mini Kit (Qiagen, Germany) according to the manufacturer's instructions, and the eluted RNA was purified using RNase-free H_2_O. The reverse transcription reaction was carried out using QuantiTect Reverse Transcription Kit (Qiagen, Germany). A SYBR Green assay was performed using primers targeted out of the designed region for dsRNA production of either ACT1 or ACT2 (Supplementary Table 1), relative to GAPDH and EF-2 as housekeeping genes, validated in a previous study^87^.

### Actin inhibition assay

To assess the impact of actin inhibition causing ablation of cell motility (Hartigan et al. 2016) on *ACT1* and *ACT2* gene expression profiles, an in vitro assay was conducted using Cytochalasin-D (Cyto-D, Sigma-Aldrich, USA, Cas No: C8273), a pharmacological inhibitor of actin polymerization, in *Sphaerospora* cells. Specifically, the cells were cultured in a medium, previously described, that contained Cyto-D at a concentration of 0.1 µM/mL for a period of 24 h (cytochalasin D-treated cells, Cyto-D). To evaluate the effect of Cyto-D removal on the cells, the cells were subsequently washed twice at 30 min and 24 h after incubation at 800*g* for 10 min using RMPI 1640 medium (removal of cytochalasin D, CytoD/-). The cells were then harvested and fixed in ECL fixation buffer (Qiagen, Germany). Total RNA was extracted from the fixed cells, and RT-qPCR was performed as described above.

### Western blotting

For the protein determination during RNAi experiments, we inspected the abundance of ACT1 via western blotting. Parasite whole-cell lysates were mixed with 2 × Laemmli sample buffer containing 10% 2-β-mercaptoethanol and heated to 97 °C for 5 min before loading onto a 12% (w/v) TGX FastCast SDS/PAGE gel. Blotted membranes were then blocked in 5% skimmed milk in PBST at room temperature for 1 h and incubated overnight with anti-*S. molnari* actin 1 primary antibody^28^ at a dilution of 1:100. The membranes were then incubated with goat anti-rabbit horseradish peroxidase-coupled antibody at a dilution of 1:5000 using the same blocking solution as the primary antibody. The signal was detected by chemiluminescence using the Clarity Western ECL Substrate. To re-use the membranes for protein detection, they were stripped using a stripping buffer (1.5 g glycine, 0.1 g sodium dodecyl sulfate, 1 mL Tween 20, and distilled H_2_O for a total of 100 mL) and re-probed according to the Abcam protocol (https://www.abcam.com/protocols/western-blot-membrane-stripping-for-restaining-protocol). As loading control, a custom-made anti-GADPH rabbit polyclonal antibody (KPLDVPSKIGERCGRSA; Davids Biotechnologie GmbH (Germany), Supplementary Fig. 5) was used at a dilution of 1:100.

### SEM microscopy

Scanning electron microscopy was performed to visualize *S. molnari* blood stages, following the methods described by^28^. The cells were first fixed in 2.5% glutaraldehyde in 0.1 M phosphate buffer at 4 °C for 1 h. After washing, the cells were adhered to coverslips with poly-D-lysine (0.1%) for 30 min, followed by further fixation in 2.5% glutaraldehyde for 15 min. The cells were then post-fixed with 1% OsO_4_, washed in distilled water, and dehydrated in a graded acetone series (5 min at each step) before critical point drying. The coverslips were mounted on stubs, gold-sputtered, and examined using a FeG-SEM JEOL 7401 F microscope.

### Statistical analyses

Differences among protocols and treatments were statistically analyzed in R (R Core Team, v. 4.2.1), using linear models (LM, package lme4 v1.1-30), using log-transformed response variables, and followed, when necessary, by post-hoc comparisons (Tuckey multiple comparison, package *multcomp* v1.4-20^88^). Specific information on the comparisons is provided in the corresponding section. When results are provided, *est* stands for an estimate of the model, and SE for standard error. Data were graphically displayed with *ggplot2* (v3.3.6^89^). In the boxplots, black horizontal lines represent the median, boxes represent 50% of the values, and upper and lower whiskers represent values > 75th and < 25th percentiles. Significant differences between groups are indicated in the figures with an asterisk, following ∗p < 0.05, ∗∗p < 0.01, ∗∗∗p < 0.001.

### Supplementary Information

Supplementary Information.

Supplementary Video 1.

Supplementary Video 2.

Supplementary Video 3.

Supplementary Video 4.

Supplementary Video 5.

Supplementary Video 6.

Supplementary Video 7.

Supplementary Video 8.

## Data Availability

The data supporting this manuscript are available in Supplementary materials and upon request from the corresponding author.
