# Supplementary material for: RNAi-directed knockdown in the cnidarian fish blood parasite Sphaerospora molnari
Source: Sci Rep. 2024 Feb 12;14:3545. doi: 10.1038/s41598-024-54171-0 (PMC10861503; doi:10.1038/s41598-024-54171-0)
Supplement: Supplementary file 1 — Supplementary Information. [file 41598_2024_54171_MOESM1_ESM.pdf]

Supplementary information for
RNAi-directed knockdown in cnidarian fish blood parasite *Sphaerospora molnari*.

Jiří Kyslík [1]*, Ana Born-Torrijos[1,2], Astrid S. Holzer[1,3], Anush Kosakyan[1,4,5]

[1] Institute of Parasitology, Biology Centre, Academy of Sciences of the Czech Republic, Ceske Budejovice, Czech Republic

[2] Department of Coastal Systems, NIOZ Royal Netherlands Institute for Sea Research, PO Box 59, 1790 AB Den Burg, Texel, The Netherlands

[3] Fish Health Division, University of Veterinary Medicine, Vienna, Austria

[4] Department of Life Sciences, University of Modena and Reggio Emilia, Modena, Italy

[5] National Biodiversity Future Center (NBFC), Palermo, Italy

***** corresponding author

Contents

Supplementary Figure 1: Nucleotide and amino acid sequences of *S. molnari* actin genes used in this study.

Supplementary Figure 2: Inconsistent gene silencing of *ACT1* and *ACT2* in *S. molnari* blood stages compared to untreated negative control (NC) over 6 h.

Supplementary Figure 3: Relative gene expression of *ACT1* knockdown in *S. molnari* blood stages compared to untreated negative control (NC) over 62 hours.

Supplementary Figure 4: Agarose gel electrophoresis of pkpl1 plasmid cleaved by using restriction enzymes (XbaI/AbaI).

Supplementary Figure 5: Amino acid sequence of *S. molnari* GAPDH used for antibody production.

Supplementary Figure 6: Original full-length blot for Figure 1c.

Supplementary Figure 7: Original full-length blot for Figure 2a.

Supplementary Figure 8: Original full-length blots for Figure 3e.

Supplementary Figure 9: Original full-length blot for Supplementary Figure 4.

Supplementary Table 1: List of primers used in this study.

Supplementary video 1: Knockdown of *ACT1* in *S. molnari* cells after 48 hours.

Supplementary video 2: Untreated control of *S. molnari* cells after 48 hours.

Supplementary video 3: Non-target GFP control of S. molnari cells after 48 hours.

Supplementary video 4: Knockdown of *ACT1* in *S. molnari* cells after 24 hours.

Supplementary video 5: Non-target GFP control of *S. molnari* cells after 24 hours.

Supplementary video 6: Untreated control of *S. molnari* cells after 24 hours.

Supplementary video 7: Knockdown of *ACT2* in *S. molnari* cells after 24 hours.

Supplementary video 8: Knockdown of *ACT2* in *S. molnari* cells after 48 hours.

Supplementary Figure 1

Nucleotide and amino acid sequences of *S. molnari* actin genes used in this study. Underlined regions indicate target sequences used for synthesis of dsRNA template.

Sphaerospora molnari _ACT1

ATGGGAGAAGAAGAGCCAGCAGCATTAGTTGTTGATAATGGATCTGGTATGTGCAA
AGCTGGTTTCGCCGGTGATGACGCACCAAGAGCTGTATTCCCCTCACTTGTAGGAAG
ACCCAGACATCAAGGTGTCATGGTTGGTATGGGACAGAAAGAATCATACGTTGGAG
ATGAAGCCCAATCCAAGAGAGGTATCTTGACCCTGAAATATCCCATTGAGCACGGT
ATTGTGACCAACTGGGATGATATGGAAAAGATCTGGCATCACACCTTCTTCAATGAG
CTCCGTGTTTCTCCAGAAGAACACCAATGTATGCTCACTGAAGCCCAATGAACCCAA
AGAACAACAGAGAAAAGATGGTTCAAATTATGTTCGATACCTTCAATCTGCCCGCA
ATGTACGTTGCTATCCAGGCTGTCCTGTCCCTGTATGCCTCTGGTAGAACCACCGGT
ATTGTGATGGACTCTGGTGATGGTGTGTCTCACACAGTCCCCATCTATGAAGGTTAC
GCTCTTCCTCACGCCATCCTCAGATTGGATTTGGCCGGTAGAGATCTCACTGAATAC
CTCATGAAGTTGCTCACCGAACGTGGAAACACCTTCACCACCTCAGCCGAAAAAGA
AATCGCCAGAGATATCAAAGAGAAACTCTGCTACGTTGCTTTGGACTTTGAACAAGA
AATGGCTAGCGCCTCCTCCTCCAGCTCATTGGAAAAACCCTATGAGATGCCCGACGG
ACAGGTTATCACCATCGGTAGTGAACGTTTCAGATGCCCAGAAGCTCTCTTCAAGCC
CAGCCTTTTGGGTAAGGAAATCAAAGGAATCCACGAGACCACCTTCGATTCCATCTC
TGCTTGCGATGTCGATATCAGAAAAGATCTGTACGCCAACACCGTCCTTTCTGGTGG
TACTACTATGTACATAGGTATTGCCGACAGAATGACCAAAGATATCACCGGTTTGGC
TGCTGCTACCATGAAGATTAAAGTTATTGCCCCACCCGAGAGAAAATACTCCGTCTG

GATTGGAGGTTCCATCCTTGCTTCCCTATCCACCTTCAACAGCATGTGGATCACCAA
ACAAGAATACGACGAGTCAGGTCCTTCCATTGTTCATCGCAAGTGCTTCTAG

Sphaerospora molnari _ACT1
MGEEEPAALVVDNGSGMCKAGFAGDDAPRAVFPSLVGRPRHQGVMVGMGQKESYVG
DEAQSKRGILTLKYPIEHGIVTNWDDMEKIWHHTFFNELRVSPEEHQCMLTEAPMNPKN
NREKMVQIMFDTFNLPAMYVAIQAVLSLYASGRTTGIVMDSGDGVSHTVPIYEGYALP
HAILRLDLAGRDLTEYLMKLLTERGNTFTTSAEKEIARDIKEKLCYVALDFEQEMASASS
SSSLEKPYEMPDGQVITIGSERFRCPEALFKPSLLGKEIKGIHETTFDSISACDVDIRKDLY
ANTVLSGGTTMYIGIADRMTKDITGLAAATMKIKVIAPPERKYSVWIGGSILASLSTFNS
MWITKQEYDESGPSIVHRKCF

Sphaerospora molnari _ACT2

ATGCAAGACGCGTCCGAAGTTCTTCCAGTTGTAGTTGACAATGGTTCAGGAATGTGC
AAAGCTGGTTTCGCTGGCGATGACGCTCCTAGAGCAGTTTTTCCATCAATTGTTGGT
CGACCAAAACACCAAGGAGTTATGGTGGGTATGGGCCAAAAAGAGGCTTATGTTGG
AGATGAAGCCCAGTCCAAGCGTGGTATTCTGGCTCTCAAGTATCCGATTGAACATGG
AGTTGTGACCAATTGGGATGATATGGAAAAGATTTGGCATCATACCTTCTACAATGA
ATTGAGAATTGCTCCTGAAGAGCATGACATTCTCTTGACGGAGGCACCCTTGAACCC
AAAAGCCAACAGAGAGAAAATGACTCAAATCATGTTTGAAGTTTTCAGTATTCCGTC
TTTCTATGTTTCCATCCAGGCTGTTCTCTCCCTTTATGCTTCTGGTCGTACAACTGGA
ATCGTTATGGATTCTGGTGACGGAGTTTCGCATACCGTTCCAATCTATGAGGGATAT
GCCCTACCACATGCTGTTCAACGTCTTGATTTAGCTGGACGAGACTTGACTGAACAT
CTTACTGTTATTCTTACAGAGCGTGGATATTCCTTTACCACAACTGCCGAAAGAGAA
ATTGTTCGAGATATGAAAGAACGCCTTTGTTATGTTGCAATGGATTATGACGCAGAG
TTGCAGAGTTCTACTTCCAGTTCCAGCGTCGAAAAAAGTTACGAAATGCCAGACGGT
CAGGTTATTACCGTTGGAGCTGAACGATTCAGATGTCCCGAAGCCTTATTCCAACCA
ATGTTGATTGGTAAAGAATCTTCTGGAATCCAATCCACCACTTACGAATCCATAATG
AAGTGCGATGTTGATATTCGAAAGGATCTCTACTCCAACATCGTTCTCTCTGGTGGT
ACTACTATGTTTCCTGGTATTGCAGATCGTATGCAAAAGGAAATCGGTGCACTTGCT
CCTTCCACTATCAAGGTAAAAATAATATCTCCTCCTGAAAGAAAGTACTCCGTCTGG
ATTGGCGGCTCCATCTTGGCATCTCTGTCTACCTTCCAAAGTATGTGGATCACCAAA
AATGAATACGATGAGATCGGTCCTAGTATCGTTCACAGGAAATGCTTCTAA

Sphaerospora molnari _ ACT2

MQDASEVLPVVVDNGSGMCKAGFAGDDAPRAVFPSIVGRPKHQGVMVGMGQKEAYV
GDEAQSKRGILALKYPIEHGVVTNWDDMEKIWHHTFYNELRIAPEEHDILLTEAPLNPK
ANREKMTQIMFEVFSIPSFYVSIQAVLSLYASGRTTGIVMDSGDGVSHTVPIYEGYALPH
AVQRLDLAGRDLTEHLTVILTERGYSFTTTAEREIVRDMKERLCYVAMDYDAELQSSTS
SSSVEKSYEMPDGQVITVGAERFRCPEALFQPMLIGKESSGIQSTTYESIMKCDVDIRKDL
YSNIVLSGGTTMFPGIADRMQKEIGALAPSTIKVKIISPPERKYSVWIGGSILASLSTFQSM
WITKNEYDEIGPSIVHRK

Supplementary Figure 2

Inconsistent gene silencing of *ACT1* and *ACT2* in *S. molnari* blood stages compared to untreated negative control (NC) over 6 h. This graph shows inconsistent relative gene expression during knockdown of *S. molnari* using low yielded dsRNA purified by manufactured protocol in three independent biological replicates. X-axis: *ACT1* KD cells (treated) and negative control *ACT1* gene expression in *S. molnari* cells (untreated), Y-axis: relative gene expression values of *ACT1* (a) *and ACT2* (b). Housekeeping genes EF2 and GAPDH were used as the reference.

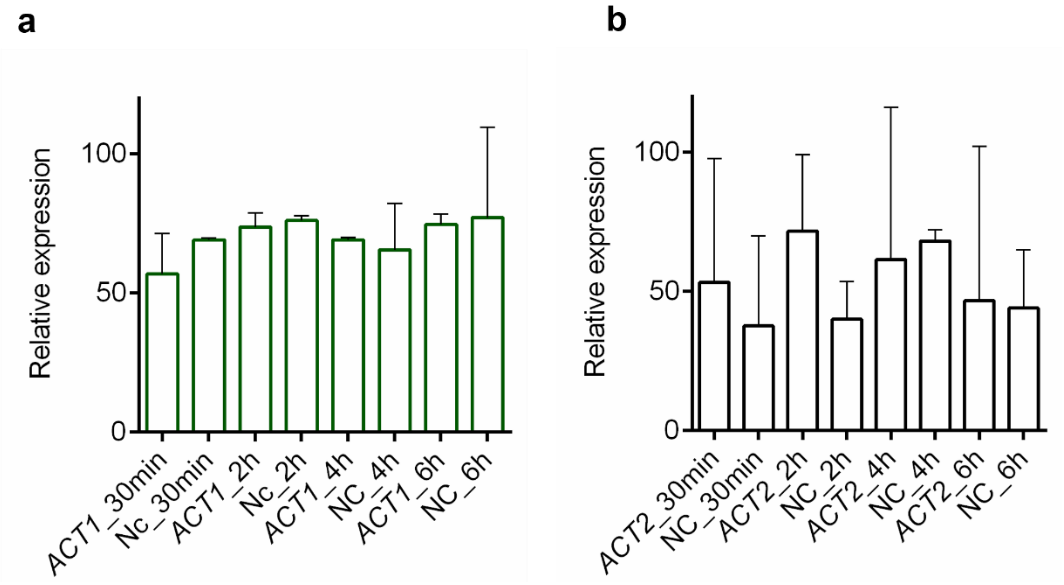

Supplementary Figure 3

Relative gene expression of *ACT1* on knockdown in *S. molnari* blood stages compared to untreated negative control (NC) over 62 hours. This graph represented by aligned dot plot shows less effective gene silencing at 62 hours of knockdown (indicated by red arrow), including the decreasing effect of gene silencing is described by the trendline (red). X-axis: *ACT1* KD cells (treated) and negative control *ACT1* gene expression in *S. molnari* cells (untreated), Y-axis: relative gene expression values of *ACT1*. Housekeeping genes EF2 and GAPDH were used as the reference.

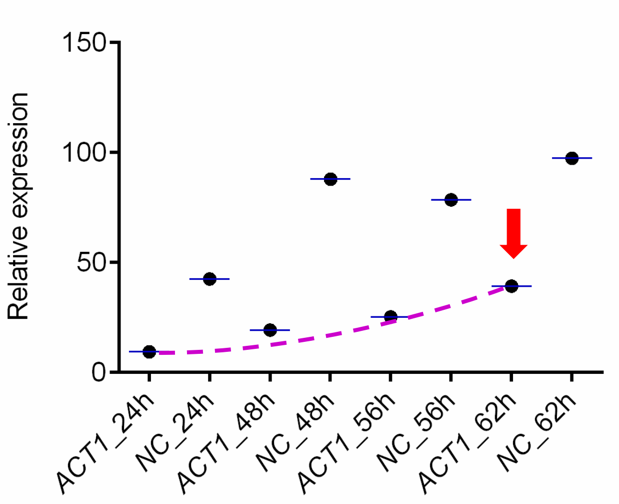

Supplementary Figure 4

Agarose gel electrophoresis of pkpl1 plasmid cleaved by using restriction enzymes (XbaI/AbaI). The figure shows digested plasmid (upper band, 3692 bp) and GFP insert (lower band, 780 bp). Original full length blot is presented in Supplementary Figure 9.

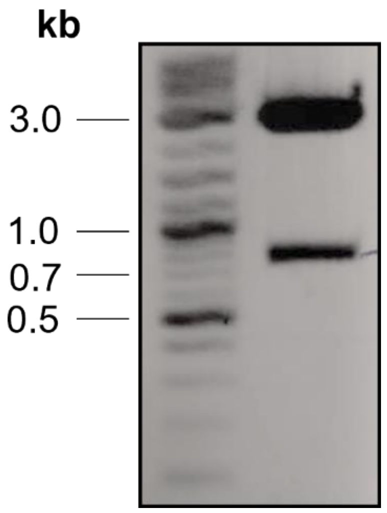

Supplementary Figure 5

Amino acid sequence of *S. molnari* GAPDH used for antibody production. Selected peptide for anti-GAPDH polyclonal antibody production is highlighted in purple.

Sphaerospora molnari _GAPDH
MGIKDSSKDLDDKQAKLSGKANQKLIWAEPTEKIRKDIPCISTMVLEASEGKQVKDGSIR
IGINGFGRIGRALMRAVIEKHLKGEKSLTVAAVNDPHMQTDTLVELLQYDSTHGRLGVP
VSHSNGQVDINKGMIVFLHLTETDPSAIHWDTNEANFIVECSGRLTTMASASKHLTQGV
KRVIISAYSKDAPLIVLGANEENYDPKTMQVVSMGSCTTNCLAPVCRILNDNFKIASVSI
TTVHSMTNSQKPLDVPSKIGERCGRSAVQNIIPYSTGAICSILRVFPELAGKINGIALRIPV
MNVSVLDLFVNLQKEVKINYLSEQLFSATWRTSTECLLLLQNHRKSVDFSELLSTMPFL
MISTQHPNLLGSIPRPVLPSIVNSTNCLSGIPTSLPFNFVFFFRYDNEYGYANRLLDMVCY
FHSREQEA

Supplementary Figure 6

Original full-length blot for Figure 1c. Adequate full-length blot is not provided due to automatic cropping of gel documentation system.

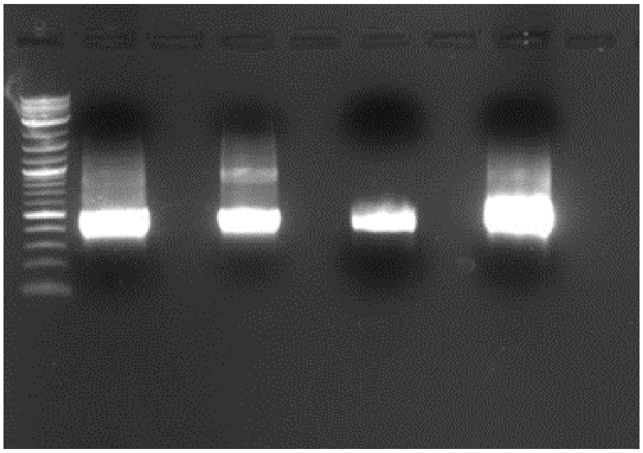

Supplementary Figure 7

Original full-length blot for Figure 2a. Adequate full-length blot is not provided due to automatic cropping of gel documentation system.

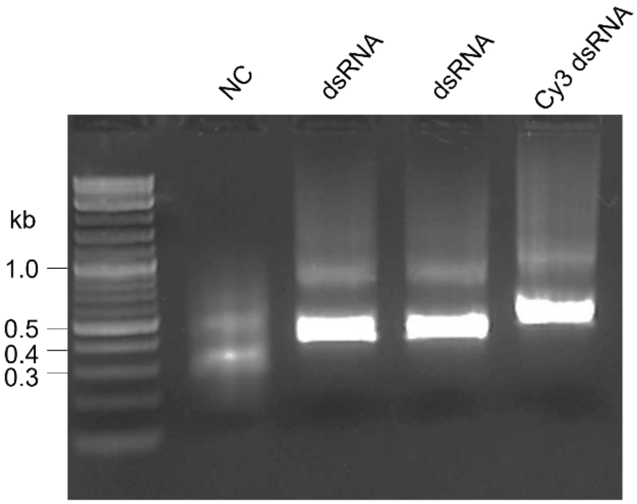

Supplementary Figure 8

Original full-length blots for Figure 3e. Composite gel blots of anti-ACT1 (a) and anti-GAPDH (d) antibodies. Negative color images of the same gels used in Figure 3e for anti-ACT1 (b) and anti-GAPDH (e) antibodies. Colorimetric snapshots of the same gels for anti-ACT1 (c) and anti-GAPDH (f) membranes, including edges. For anti-ACT1 blots, membrane edges are not visible due to default exposure settings in the ChemiDoc software (BioRad, USA). Moreover, the blots were cut prior to hybridization with antibodies; thus, gel wells are not presented.

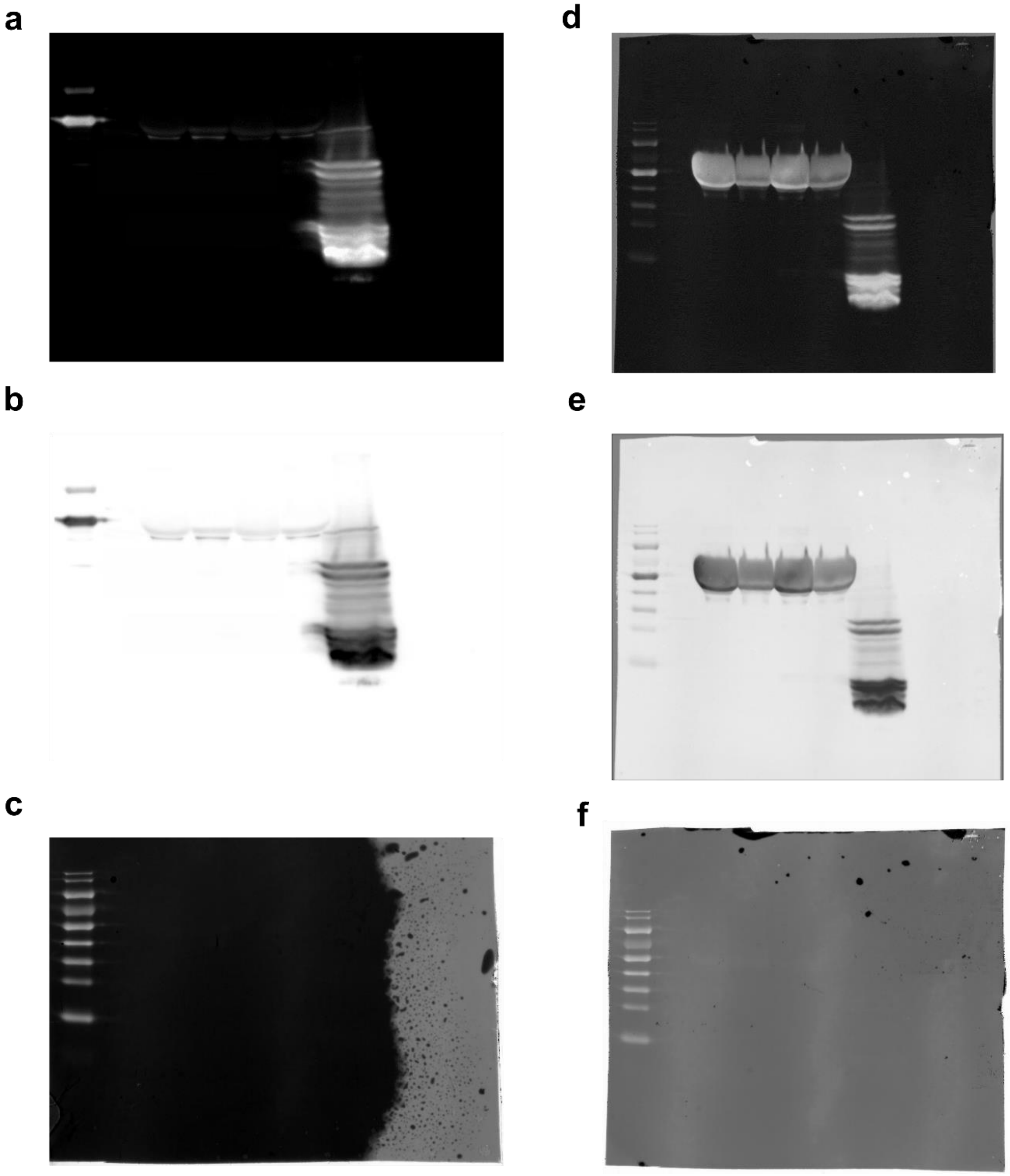

Supplementary Figure 9

Original full-length blot for Supplementary Figure 4. Gel blot of pkpl1 plasmid cleaved by using restriction enzymes (XbaI/AbaI). Adequate full-length blot is not provided due to automatic cropping of gel documentation system.

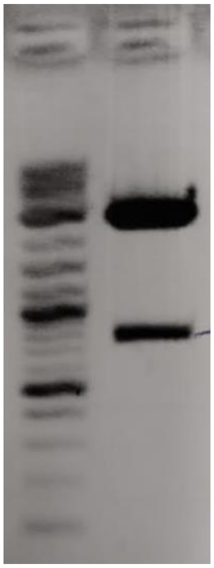

Supplementary Table 1: List of primers used in this study. Underlined sequence regions (red) indicate T7-promoter overhangs.

Name	5´-3´ primer sequence	Reference
Smol_ACT1_T7-Fwd	TAATACGACTCACTATAGGGAGATCCAGCTCATTGGAAAAACC	This study
Smol_ACT1_T7-Rev	TAATACGACTCACTATAGGGAGAGCGATGAACAATGGAAGGACC	This study
Smol_ACT2_T7-Fwd	TAATACGACTCACTATAGGGAGACCACATGCTGTTCAACGTCT	This study
Smol_ACT2_T7-Rev	TAATACGACTCACTATAGGGAGAAGTGCACCGATTTCCTTTTG	This study
Smol_GFP_T7-Fwd	TAATACGACTCACTATAGGGAGACGGCAACTACAAGACACG	This study
Smol_GFP_T7-Rev	TAATACGACTCACTATAGGGAGAGTTCATCCATGCCATGTGTA	This study
Smol_ACT1-Fwd	CCTCAGCCGAAAAAGAAATCG	This study
Smol_ACT1-Rev	GGTTTTTCCAATGAGCTGGA	This study
Smol_ACT2-Fwd	GCGTGGTATTCTGGCTCTCAAG	This study
Smol_ACT2-Rev	CCTCCGTCAAGAGAATGTCA	This study
Smol_GAPDH-Fwd	TATCGACCTGGCCGTTACTG	Kosakyan et al. 2019
Smol_GAPDH-Rev	GTTGCTGCTGTCAATGACCC	Kosakyan et al. 2019
Smol_EF2-Fwd	TCCGGCAGGCAAGAAGGTTT	Kosakyan et al. 2019
Smol_EF2-Rev	CCAAGTTGGATACGGATTACGAGT	Kosakyan et al. 2019

Supplementary video 1: Knockdown of *ACT1* in *S. molnari* cells after 48 hours.

Recorded motility of *S. molnari* blood stages during knockdown of *ACT1* after 48 hours (*ACT1*_48h) showing loss of axial motility with continuous forming of membrane folds, Scale bar – 5 µm.

Supplementary video 2: Untreated control of *S. molnari* cells after 48 hours.

Recorded motility of untreated *S. molnari* blood stages (untreated control) after 48 hours (*NC*_48h) showing normal membrane fold induced tumbling motility, Scale bar – 5 µm.

Supplementary video 3: Non-target GFP control of *S. molnari* cells after 48 hours.

Recorded motility of knockdown of non-target GFP *S. molnari* blood stages after 48 hours (*GFP*_48h) showing normal membrane fold induced tumbling motility, Scale bar – 5 µm.

Supplementary video 4: Knockdown of *ACT1* in *S. molnari* cells after 24 hours.

Recorded motility of *S. molnari* blood stages during knockdown of *ACT1* after 24 hours (*ACT1*_24h) showing normal membrane fold induced tumbling motility, Scale bar – 5 µm.

Supplementary video 5: Non-target GFP control of *S. molnari* cells after 24 hours.

Recorded motility of knockdown of non-target GFP *S. molnari* blood stages after 24 hours (*GFP*_24h) showing normal membrane fold induced tumbling motility, Scale bar – 5 µm.

Supplementary video 6: Untreated control of *S. molnari* cells after 24 hours.

Recorded motility of untreated *S. molnari* blood stages (untreated control) after 24 hours (*NC*_24h) showing normal membrane fold induced tumbling motility, Scale bar – 5 µm.

Supplementary video 7: Knockdown of *ACT2* in *S. molnari* cells after 24 hours.

Recorded motility of *S. molnari* blood stages during knockdown of *ACT2* after 48 hours (*ACT2*_48h) showing normal membrane fold induced tumbling motility, Scale bar – 5 µm.

Supplementary video 8: Knockdown of *ACT2* in *S. molnari* cells after 48 hours.

Recorded motility of *S. molnari* blood stages during knockdown of *ACT2* after 48 hours (*ACT2*_48h) showing normal membrane fold induced tumbling motility, Scale bar – 5 µm.

References

Kosakyan, A. *et al*. Selection of suitable reference genes for gene expression studies in myxosporean (Myxozoa, Cnidaria) parasites. *Sci Rep*. **9**, 15073 (2019).
